# Practical challenges for functional validation of *STAT1* gain of function genetic variants

**DOI:** 10.1093/cei/uxad008

**Published:** 2023-02-01

**Authors:** Adriana S Albuquerque, Jesmeen Maimaris, Alexander J McKenna, Jonathan Lambourne, Fernando Moreira, Sarita Workman, Karyn Megy, Ilenia Simeoni, Hana Lango Allen, NIHR BioResource-Rare Disease Consortium, Zoe Adhya, Hana Alachkar, Ariharan Anantharachagan, Richard Antrobus, Gururaj Arumugakani, Chiara Bacchelli, Helen Baxendale, Claire Bethune, Shahnaz Bibi, Barbara Boardman, Claire Booth, Michael Browning, Mary Brownlie, Siobhan Burns, Anita Chandra, Hayley Clifford, Nichola Cooper, Sophie Davies, John Dempster, Lisa Devlin, Rainer Doffinger, Elizabeth Drewe, David Edgar, William Egner, Tariq El-Shanawany, Bobby Gaspar, Rohit Ghurye, Kimberley Gilmour, Sarah Goddard, Pavel Gordins, Sofia Grigoriadou, Scott Hackett, Rosie Hague, Lorraine Harper, Grant Hayman, Archana Herwadkar, Stephen Hughes, Aarnoud Huissoon, Stephen Jolles, Julie Jones, Peter Kelleher, Nigel Klein, Taco Kuijpers, Dinakantha Kumararatne, James Laffan, Hana Lango Allen, Sara Lear, Hilary Longhurst, Lorena Lorenzo, Jesmeen Maimaris, Ania Manson, Elizabeth McDermott, Hazel Millar, Anoop Mistry, Valerie Morrisson, Sai Murng, Iman Nasir, Sergey Nejentsev, Sadia Noorani, Eric Oksenhendler, Mark Ponsford, Waseem Qasim, Ellen Quinn, Isabella Quinti, Alex Richter, Crina Samarghitean, Ravishankar Sargur, Sinisa Savic, Suranjith Seneviratne, Carrock Sewall, Fiona Shackley, Ilenia Simeoni, Kenneth G C Smith, Emily Staples, Hans Stauss, Cathal Steele, James Thaventhiran, Moira Thomas, Adrian Thrasher, Steve Welch, Lisa Willcocks, Sarita Workman, Austen Worth, Nigel Yeatman, Patrick Yong, Sofie Ashford, John Bradley, Debra Fletcher, Tracey Hammerton, Roger James, Nathalie Kingston, Willem Ouwehand, Christopher Penkett, F Lucy Raymond, Kathleen Stirrups, Marijke Veltman, Tim Young, Sofie Ashford, Matthew Brown, Naomi Clements-Brod, John Davis, Eleanor Dewhurst, Marie Erwood, Amy Frary, Rachel Linger, Jennifer Martin, Sofia Papadia, Karola Rehnstrom, William Astle, Antony Attwood, Marta Bleda, Keren Carss, Louise Daugherty, Sri Deevi, Stefan Graf, Daniel Greene, Csaba Halmagyi, Matthias Haimel, Fengyuan Hu, Roger James, Hana Lango Allen, Vera Matser, Stuart Meacham, Karyn Megy, Christopher Penkett, Olga Shamardina, Kathleen Stirrups, Catherine Titterton, Salih Tuna, Ernest Turro, Ping Yu, Julie von Ziegenweldt, Abigail Furnell, Rutendo Mapeta, Ilenia Simeoni, Simon Staines, Jonathan Stephens, Kathleen Stirrups, Deborah Whitehorn, Paula Rayner-Matthews, Christopher Watt, Emma C Morris, Siobhan O Burns

**Affiliations:** Institute of Immunity and Transplantation, University College London, London, UK; Institute of Immunity and Transplantation, University College London, London, UK; Department of Immunology, Royal Free London NHS Foundation Trust, London, UK; Institute of Immunity and Transplantation, University College London, London, UK; Department of Infectious Diseases, Royal London Hospital, Barts Health NHS Trust, London, UK; Department of Immunology, Royal Free London NHS Foundation Trust, London, UK; Department of Immunology, Royal Free London NHS Foundation Trust, London, UK; NIHR BioResource-Rare Disease Consortium, Cambridge University Hospitals, Cambridge Biomedical Campus, Cambridge, UK; NIHR BioResource-Rare Disease Consortium, Cambridge University Hospitals, Cambridge Biomedical Campus, Cambridge, UK; NIHR BioResource-Rare Disease Consortium, Cambridge University Hospitals, Cambridge Biomedical Campus, Cambridge, UK; Cambridge Genomics Laboratory, Cambridge University Hospitals NHS Foundation Trust, Cambridge, UK; Institute of Immunity and Transplantation, University College London, London, UK; Department of Immunology, Royal Free London NHS Foundation Trust, London, UK; Institute of Immunity and Transplantation, University College London, London, UK; Department of Immunology, Royal Free London NHS Foundation Trust, London, UK

**Keywords:** STAT1, gain of function, variants of unknown significance, flow cytometry, primary immunodeficiency, chronic mucocutaneous candidiasis

Wider application of next generation genetic sequencing (NGS) has significantly improved diagnosis for patients with inborn errors of immunity (IEI) and is increasingly a routine part of clinical practice [1, 2]. However, functional validation of genetic variants of unknown significance (VUS) remains a significant challenge for confirming definitive diagnoses, both due to inconsistent access to specialized testing and poor standardization of currently available assays [3]. The challenges are greater for conditions where clinical phenotype is not confirmatory due to heterogeneity or incomplete penetrance.

*STAT1* gain-of-function (GOF) immunodeficiency typically presents with chronic mucocutaneous candidiasis (CMC), with or without a combination of bacterial, viral or mycobacterial infections, along with other complications including bronchiectasis, autoimmunity and vascular abnormalities [4, 5]. Over 75 different individual genetic mutations have been described to confer gain of function in the *STAT1* gene [6] and classically result in increased phosphorylation of STAT1 (pSTAT1) in immune cells, either at baseline or following cytokine stimulation [7]. Therefore, most immunology laboratories that offer validation assays for *STAT1* VUS rely on comparing pSTAT1 levels between a potential index case and one or a group of healthy controls. However, levels of pSTAT1 at baseline and upregulation after cytokine stimulation vary considerably in the healthy population and overlap with levels observed in STAT1 GOF patients.

In this study, we performed technically standardized flow cytometry assays using peripheral blood obtained from six patients with five different *STAT1* VUS, identified through whole genome or targeted chip panel sequencing for suspected IEI (see Supplementary Table 1 and Methods). We compared these against 4 patients with known pathogenic GOF mutations in *STAT1* and 16 healthy controls. A group of six patients with Common Variable Immunodeficiency (CVID) who had no pathogenic mutations identified in currently known IEI genes by NGS and no rare VUS in *STAT1* were used as disease controls. Healthy control samples were taken on the same day as patient or disease control samples and assays performed in parallel. *STAT1* VUS tested here were rare (allele frequency <10^−4^ in reference databases) with variable prediction of deleterious impact using *in silico* prediction tools (Table 1 and Supplementary Fig. 1a and b).

**Table 1.** Predicted functional impact of *STAT1* variants evaluated by *in silico* analysis and functional assays

Group	SIFT raw (<0.05)	PolyPhen2 Raw	CADD PHRED	Patient/mutation	pSTAT1	pSTAT1	IL-17
	<0.05	>0.85	>20		Fold change > 2^*^ (normalized for HC)	>Mean + SD of HC	<HC of the day
VUS	Yes	Yes	Yes	**1/G416R**	Yes	No	Yes
	Yes	Yes	Yes	**2/F404V**	No	Yes	Yes
	No	No	No	**3/E284K**	No	Yes	Yes
	No	No	No	**4/E284K**	Yes	Yes	Yes
	Yes	Yes	Yes	**5/K344Q**	Yes	Yes	Yes
	No	No	No	**6/T419K**	Yes	Yes	Yes
GOF	Yes	Yes	Yes	**7/K388E**	Yes	Yes	Yes
	No	No	No	**8/T720I**	Yes	Yes	Yes
	Yes	Yes	Yes	**9/T385M**	Yes	Yes	Yes
	Yes	Yes	No	**10/P293S**	Yes	Yes	Yes

^*^Normalized for the healthy control of the day; VUS = variant of unknown significance; GOF = gain of function; HC= healthy control; SD = standard deviation.

Significant variability was seen in STAT1 levels and in pSTAT1 upregulation in response to interferon-alpha (IFN-α) stimulation for all groups (Supplementary Figs 1c-e and 2a). When considered by group, both *STAT1* GOF mutations and *STAT1* VUS showed a significant increase in upregulation of pSTAT1 compared with healthy control (Supplementary Fig. 1d), although there was an overlap in the range observed for each group. We then considered each genetic variant individually to determine whether or not VUS could be assigned as GOF. When normalized against their matched healthy control sample, all patients with GOF mutations and 4/6 with VUS demonstrated at least 2-fold higher upregulation in pSTAT1 levels after stimulation (Fig. 1a). Two patients with VUS did not achieve this arbitrary cut off and one of these had a matched healthy control at the upper limit of the healthy control range (Fig. 1b). To reduce the impact of individual healthy control variation, we compared each VUS and GOF mutation against the whole healthy control range and identified that all patients with GOF and 5/6 with VUS upregulated pSTAT1 above a cut off of mean+1SD of the healthy control range (Fig. 1c). Variant location did not appear to impact degree of upregulation (Fig. 1d). Upregulation of total STAT1 was more variable; although all GOF and VUS showed upregulation to at least an arbitrary cut-off of 1.4x healthy control (Fig. 1e), this was considered to be not sufficiently robust to assist diagnosis (Supplementary Fig. 1e). CVID disease controls as a group behaved like healthy controls, with comparable upregulation of pSTAT1 and STAT1 (Fig. 1a, c–e, and Supplementary Fig. 1c–e). To test inclusion of other parameters that could improve diagnosis of GOF mutations, we quantified the frequency of CD4+ T cells producing IL-17 (Fig. 1f, Supplementary Figs 1f and 2b) and upregulation of the expression of STAT1-inducible CXCL-10 in monocytes (Supplementary Figs 3 and 4). In keeping with the known suppression of STAT3 signalling in *STAT1* GOF [8], all known GOF mutations supported reduced frequency of Th17 cells (Fig. 1f; Supplementary Figs 1f and 2b). All patients with *STAT1* VUS also had lower levels of Th17+ cells than the matched healthy control samples analysed on the same day. In contrast, CXCL-10 expression by monocytes at baseline and fold-change in levels following stimulation were variable between known GOF mutations (Supplementary Fig. 3), reducing the utility of this assay for functional validation of *STAT1* VUS.

**Figure 1.**
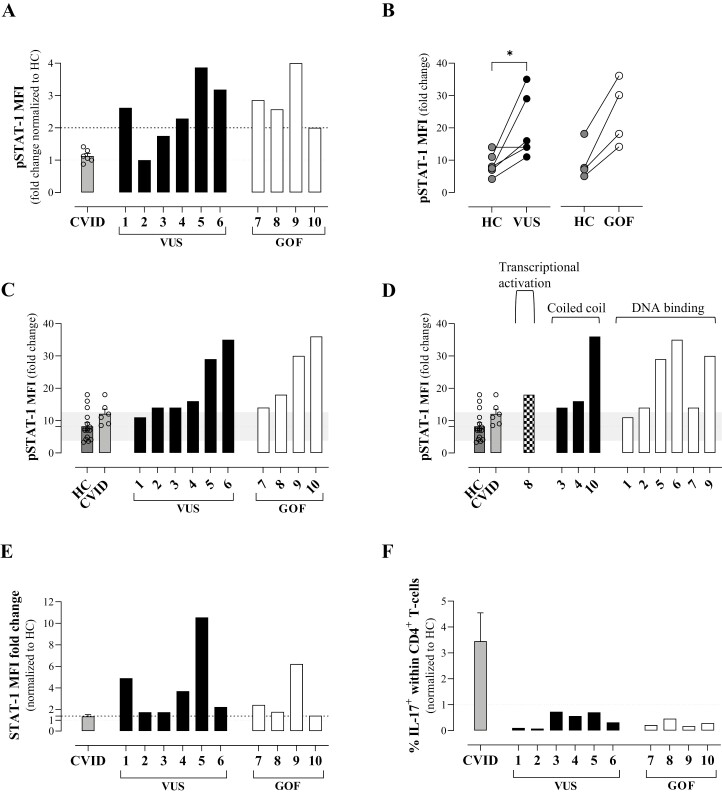
STAT1 phosphorylation and Th17 cells. Levels of expression of pSTAT1 (**a**–**d**) or total STAT1 (**e**) on gated CD3+ T cells after stimulation with IFN-α. (**f**) Frequency of IL-17+ cells within CD4+ lymphocytes. Fold change was calculated as the MFI ratio between IFN-α-stimulated and non-stimulated cells. Each dot or number represents one individual. For (b and c), the mean of the HC group is shown as a dotted line and the standard deviation of the HC group as grey shading.

To support diagnostic reporting, we considered a combination of pSTAT1 and IL-17 production results for each patient (Table 1). All GOF mutations demonstrated elevated levels of pSTAT1 in response to IFN-α stimulation (at least 2-fold upregulation compared with the matched healthy control and above the mean+1SD of the healthy control group range) plus had a frequency of Th17 cells below the matched healthy control. All six patients with VUS also demonstrated increased upregulation of pSTAT1, either when considered against the matched healthy control of the day or against the healthy control group range (Fig. 1a–d) and all 6 had low levels of Th17 compared with the control (Fig. 1f). Thus, using this combination of pSTAT1 and IL-17 measurements, we concluded that all 5 VUS in 6 patients were GOF.

This study highlights the difficulty of definitively assigning GOF status to *STAT1* VUS using the single assessment of pSTAT1 and the benefit of including Th17 quantification as an additional parameter. For laboratories performing pSTAT1 assays, highly standardized phosflow protocols and flow cytometer settings together with a local healthy control range are important to reduce variation and improve assessment of index cases. In our hands, pSTAT1 assays only perform reproducibly on fresh blood (taken same day) and older samples are not reliably interpretable. To mitigate against healthy control variation, we suggest that assessment of pSTAT1 is made both against a same-day matched control and the local healthy control range. Future refinement of functional assessment for *STAT1* VUS requires development of additional robust assays that can be translated from research to diagnostic laboratories.

## Supplementary Material

uxad008_suppl_Supplementary_Data_S1

uxad008_suppl_Supplementary_Data_S2

uxad008_suppl_Supplementary_Figures

uxad008_suppl_Supplementary_Table_S1

## Data Availability

The datasets used and analysed in the current study are available from the corresponding author on reasonable request.

## Abbreviations

CMC: chronic mucocutaneous candidiasis
CVID: common variable immunodeficiency
CXCL-10: C-X-C motif chemokine ligand 10
GOF: gain of function
HC: healthy control
IEI: inborn errors of immunity
IFN-α: interferon-alpha
MFI: mean fluorescence intensity
NGS: next generation genetic sequencing
PBMC: peripheral blood mononuclear cell
PID: primary Immunodeficiency
PMA: phorbol 12-myristate 13-acetate
SD: standard deviation
pSTAT1: phosphorylation of signal transducer and activator of transcription 1
STAT1: signal transducer and activator of transcription 1
VUS: variant of unknown significance.

## Consortia

NIHR BioResource – Rare Diseases Consortium:

The members of the NIHR BioResource – Rare Diseases PID Consortium are Zoe Adhya, Hana Alachkar, Ariharan Anantharachagan, Richard Antrobus, Gururaj Arumugakani, Chiara Bacchelli, Helen Baxendale, Claire Bethune, Shahnaz Bibi, Barbara Boardman, Claire Booth, Michael Browning, Mary Brownlie, Siobhan Burns, Anita Chandra, Hayley Clifford, Nichola Cooper, Sophie Davies, John Dempster, Lisa Devlin, Rainer Doffinger, Elizabeth Drewe, David Edgar, William Egner, Tariq El-Shanawany, Bobby Gaspar, Rohit Ghurye, Kimberley Gilmour, Sarah Goddard, Pavel Gordins, Sofia Grigoriadou, Scott Hackett, Rosie Hague, Lorraine Harper, Grant Hayman, Archana Herwadkar, Stephen Hughes, Aarnoud Huissoon, Stephen Jolles, Julie Jones, Peter Kelleher, Nigel Klein, Taco Kuijpers (principal investigator), Dinakantha Kumararatne, James Laffan, Hana Lango Allen, Sara Lear, Hilary Longhurst, Lorena Lorenzo, Jesmeen Maimaris, Ania Manson, Elizabeth McDermott, Hazel Millar, Anoop Mistry, Valerie Morrisson, Sai Murng, Iman Nasir, Sergey Nejentsev, Sadia Noorani, Eric Oksenhendler, Mark Ponsford, Waseem Qasim, Ellen Quinn, Isabella Quinti, Alex Richter, Crina Samarghitean, Ravishankar Sargur, Sinisa Savic, Suranjith Seneviratne, Carrock Sewall, Fiona Shackley, Ilenia Simeoni, Kenneth G. C. Smith (principal investigator), Emily Staples, Hans Stauss, Cathal Steele, James Thaventhiran, Moira Thomas, Adrian Thrasher (principal investigator), Steve Welch, Lisa Willcocks, Sarita Workman, Austen Worth, Nigel Yeatman, and Patrick Yong.

The members of the NIHRBR-RD Management Team are Sofie Ashford, John Bradley, Debra Fletcher, Tracey Hammerton, Roger James, Nathalie Kingston, Willem Ouwehand, Christopher Penkett, F Lucy Raymond, Kathleen Stirrups, Marijke Veltman, and Tim Young.

The members of the NIHRBR-RD Enrolment and Ethics Team are Sofie Ashford, Matthew Brown, Naomi Clements-Brod, John Davis, Eleanor Dewhurst, Marie Erwood, Amy Frary, Rachel Linger, Jennifer Martin, Sofia Papadia, and Karola Rehnstrom.

The members of the NIHRBR-RD Bioinformatics Team are William Astle, Antony Attwood, Marta Bleda, Keren Carss, Louise Daugherty, Sri Deevi, Stefan Graf, Daniel Greene, Csaba Halmagyi, Matthias Haimel, Fengyuan Hu, Roger James, Hana Lango Allen, Vera Matser, Stuart Meacham, Karyn Megy, Christopher Penkett, Olga Shamardina, Kathleen Stirrups, Catherine Titterton, Salih Tuna, Ernest Turro, Ping Yu, and Julie von Ziegenweldt.

The members of the Cambridge Translational Genomics Laboratory are Abigail Furnell, Rutendo Mapeta, Ilenia Simeoni, Simon Staines, Jonathan Stephens, Kathleen Stirrups, Deborah Whitehorn, Paula Rayner-Matthews, and Christopher Watt.
