# Supplementary material for: Practical challenges for functional validation of STAT1 gain of function genetic variants
Source: Clin Exp Immunol. 2023 Feb 1;212(2):166–9. doi: 10.1093/cei/uxad008 (PMC10128160; doi:10.1093/cei/uxad008)
Supplement: uxad008_suppl_Supplementary_Data_S1 [file uxad008_suppl_supplementary_data_s1.docx]

**Supplementary Figures legend**

**Supplementary Figure 1 – *STAT1* variant pathogenicity, location, levels and phosphorylation (a)** In-silico prediction and **(b)** location of *STAT1* variants. **c)** Mean expression of STAT1 in all groups studied. **d)** pSTAT1 upregulation in CD3+ T cells after stimulation with IFN-α. **e)** STAT1 expression in each VUS or GOF individuals. **f)** Frequency of CD4+ T cell producing IL-17 after PMA+I stimulation in VUS and GOF group of patients compared to the HC of the day. Each dot represents one individual. For e) the mean of the HC group is shown as a dotted line and the standard deviation of the HC group as grey shading.

**Supplementary Figure 2 – Flow cytometry gating strategy for the analysis of STAT1 and pSTAT1 expression and Th17 cells.** Representative FACS plots showing the gating strategy to analyse **(a)** the expression of STAT1 and pSTAT1 within CD3+ cells and **(b)** the frequency of CD4+ T cell producing IL-17 after PMA+I stimulation in HC, CVID, VUS and GOF group of patients. Numbers inside dot plots represent percentage of cells and numbers inside histograms represent MFI.

**Supplementary Figure 3 –** Monocyte CXCL-10 expression before **(a-b)** and after **(c-d)** stimulation with IFN-α. Fold change calculated as MFI ratio between IFNα-stimulated and non-stimulated cells. For a-d) the mean of the HC group is shown as a dotted line and the standard deviation of the HC group as grey shading.

**Supplementary Figure 4 – Flow cytometry gating strategy for the analysis of CXCL-10 expression on monocytes.** Representative FACS plots showing the gating strategy to analyse the expression of CXCL-10 within CD14+ cells after stimulation with IFN-α in HC, CVID, VUS and GOF group of patients. Numbers inside dot plots represent percentage of cells and numbers inside histograms represent MFI.
