# Supplementary material for: Practical challenges for functional validation of STAT1 gain of function genetic variants
Source: Clin Exp Immunol. 2023 Feb 1;212(2):166–9. doi: 10.1093/cei/uxad008 (PMC10128160; doi:10.1093/cei/uxad008)
Supplement: uxad008_suppl_Supplementary_Data_S2 [file uxad008_suppl_supplementary_data_s2.docx]

**Supplementary Methods**

**Study design and patients**

Patients with typical features of chronic mucocutaneous candidiasis (CMC) with *STAT1* variants were identified from 2 tertiary immunology services in London, UK. Patients with *STAT1* variants not previously described in the literature were categorized as variants of unknown significance (VUS). These patients were compared with 4 CMC patients with known pathogenic *STAT1* gain of function variants, referred to in the paper as STAT1 GOF, a group of six patients with a diagnosis of common variable immunodeficiency (CVID) identified at one of the London centers and sixteen healthy controls (HC). All assays were performed on freshly collected blood from patients and a healthy control sample was done in parallel.

**Genetic analysis**

All patients, including CVID disease controls underwent next generation genetic sequencing - either whole genome sequencing in the NIHR Bioresource – Rare Disease research study^1^ or targeted chip panel as part of the GRID study^2^.

Nine *STAT1* mutations were identified in 10 patients with CMC, of which 5 were novel mutations and 4 were previously described mutations leading to gain-of-function (GOF) phenotype. Other known causes of IEI were excluded. The predicted functional impact of the *STAT1* variants was evaluated using the *in-silico* tools SIFT^3^, PolyPhen2^4^, CADD^5^ and REVEL^6^.

CVID patients included in this study did not have any rare variation in *STAT1* or any other known causes of IEI identified (analyzed according to previously published gene lists for PID WGS^1^ and GRID targeted chip panel^2^).

**Homology modelling and mutation localisation**

Homology modelling was performed with MODELLER. To build a structure of full-length, human STAT1 protein, three PDB templates were used (1yvI.1, 1yvI.2 and 1bf5) which cover different regions and conformations of the human STAT1 protein sequence. Residue-wise alignment among the PDB templates and the human STAT1 protein sequence was prepared by referring to annotations from the structure integration with function, taxonomy and sequence (SIFTS) database. Computational analysis was performed using the PyMol molecular graphics system.

**STAT1 phosphorylation and total STAT1 quantification by flow cytometry**

pSTAT1 was quantified in whole blood immediately after collection, as previously described^7^. Whole blood was stimulated with IFN-α (Merck Sharp & Dohme Limited) 50000U/ml for 15min and immediately lysed/fixed with Lyse/Fix buffer (BD Biosciences). Cells were then permeabilized with Perm III (BD Biosciences) for 30min at 4°C and stained for 1h at room temperature with anti-human anti-pSTAT1 (clone 4a, BD Biosciences), anti-STAT1 (clone 1/Stat1, BD Biosciences), and anti-CD3 (clone SK7, Biolegend). pSTAT1 and STAT1 expression were analyzed within a CD3+ gate and results are shown as mean fluorescence intensity (MFI) fold change to unstimulated cells or normalized for the healthy control of the day done in parallel.

**Quantification of CXCL-10 by flow cytometry**

Peripheral blood mononuclear cells (PBMCs) were isolated from freshly collected blood by Ficoll-Paque (GE Healthcare) gradient. PBMCs were left untreated or were stimulated with 50000 IU/ml IFN-α for 4h at 37°C. After fixation with BD Cytofix/Cytoperm buffer (BD Biosciences), cells were surface stained with anti-human anti-CD3 (clone SK7, Biolegend) and anti-CD14 (clone MφP9, BD Biosciences), permeabilized with Perm buffer (BD Biosciences) and stained with anti-CXCL10 (clone IP-10, Biolegend). Data were analyzed within a CD3negCD14+ gate and results are shown as MFI at baseline or fold change to unstimulated cells.

**Cytokine production at the single-cell level**

Cytokine production was assessed at the single cell level after 4h stimulation of PBMCs with phorbol 12-myristate 13-acetate (PMA, Sigma-Aldrich) 50ng/ml plus Ionomycin (Sigma-Aldrich) 500ng/ml in the presence of Brefeldin A (Sigma-Aldrich) 10μg/ml, as previously described^8^. After stimulation, cells were surface stained with anti-human anti-CD3 (clone UCHT1, BD Biosciences) and anti-CD4 (clone SK3, BD Biosciences) followed by fixation with BD Cytofix/Cytoperm (BD Biosciences) and permeabilized with Perm/wash Buffer (BD Biosciences). Cells were intracellularly stained with anti-human anti-IL-17 (clone SCPL1362, BD Biosciences). Results are shown as frequency of CD4+ cells producing IL-17 after normalization for the healthy control done in parallel.

**Flow cytometry**

At least 150000 events were acquired on a BD LSRFortessa (BD Biosciences) and data was analyzed using FlowJo software (TreeStar). After lymphocyte or monocyte gate definition, doublets were excluded, and cells analyzed within the mentioned gates.

**Statistical analysis**

Statistical analysis was performed with GraphPad Prism Version 9.2. Data was compared using One-way ANOVA. Results were expressed as mean. *P*-values < 0.05 were considered significant.

**References**

1. Thaventhiran JED, Lango Allen H, Burren OS, Rae W, Greene D, Staples E, et al. Whole-genome sequencing of a sporadic primary immunodeficiency cohort. Nature 2020;583:90–5.

2. Simeoni I, Shamardina O, Deevi SV, Thomas M, Megy K, Staples E, et al. GRID-Genomics of Rare Immune Disorders: a highly sensitive and specific diagnostic gene panel for patients with primary immunodeficiencies. Available from: https://doi.org/10.1101/431544.

3. Sim NL, Kumar P, Hu J, Henikoff S, Schneider G, Ng PC. SIFT web server: Predicting effects of amino acid substitutions on proteins. Nucleic Acids Research. 2012;40:W452-7.

4. Adzhubei IA, Schmidt S, Peshkin L, Ramensky VE, Gerasimova A, Bork P, et al. A method and server for predicting damaging missense mutations. Nature Methods 2010; 7:248–9.

5. Kircher M, Witten DM, Jain P, O’roak BJ, Cooper GM, Shendure J. A general framework for estimating the relative pathogenicity of human genetic variants. Nature Genetics 2014;46:310–5.

6. Ioannidis NM, Rothstein JH, Pejaver V, Middha S, McDonnell SK, Baheti S, et al. REVEL: An Ensemble Method for Predicting the Pathogenicity of Rare Missense Variants. Am J Human Genet. 2016;99:877–85.

7. Silva SL, Albuquerque AS, Serra-Caetano A, Foxall RB, Pires AR, Matoso P, et al. Human naïve regulatory T-cells feature high steady-state turnover and are maintained by IL-7. Oncotarget 2016;7:12163-75.8. Albuquerque AS, Fernandes SM, Tendeiro R, Cheynier R, Lucas M, Silva SL, et al. Major CD4 T-cell depletion and immune senescence in a patient with chronic granulomatous disease. Front Immunol 2017;8:543.
