# Supplementary material for: Practical challenges for functional validation of STAT1 gain of function genetic variants
Source: Clin Exp Immunol. 2023 Feb 1;212(2):166–9. doi: 10.1093/cei/uxad008 (PMC10128160; doi:10.1093/cei/uxad008)
Supplement: uxad008_suppl_Supplementary_Figures [file uxad008_suppl_supplementary_figures.pptx]

## Slide 1
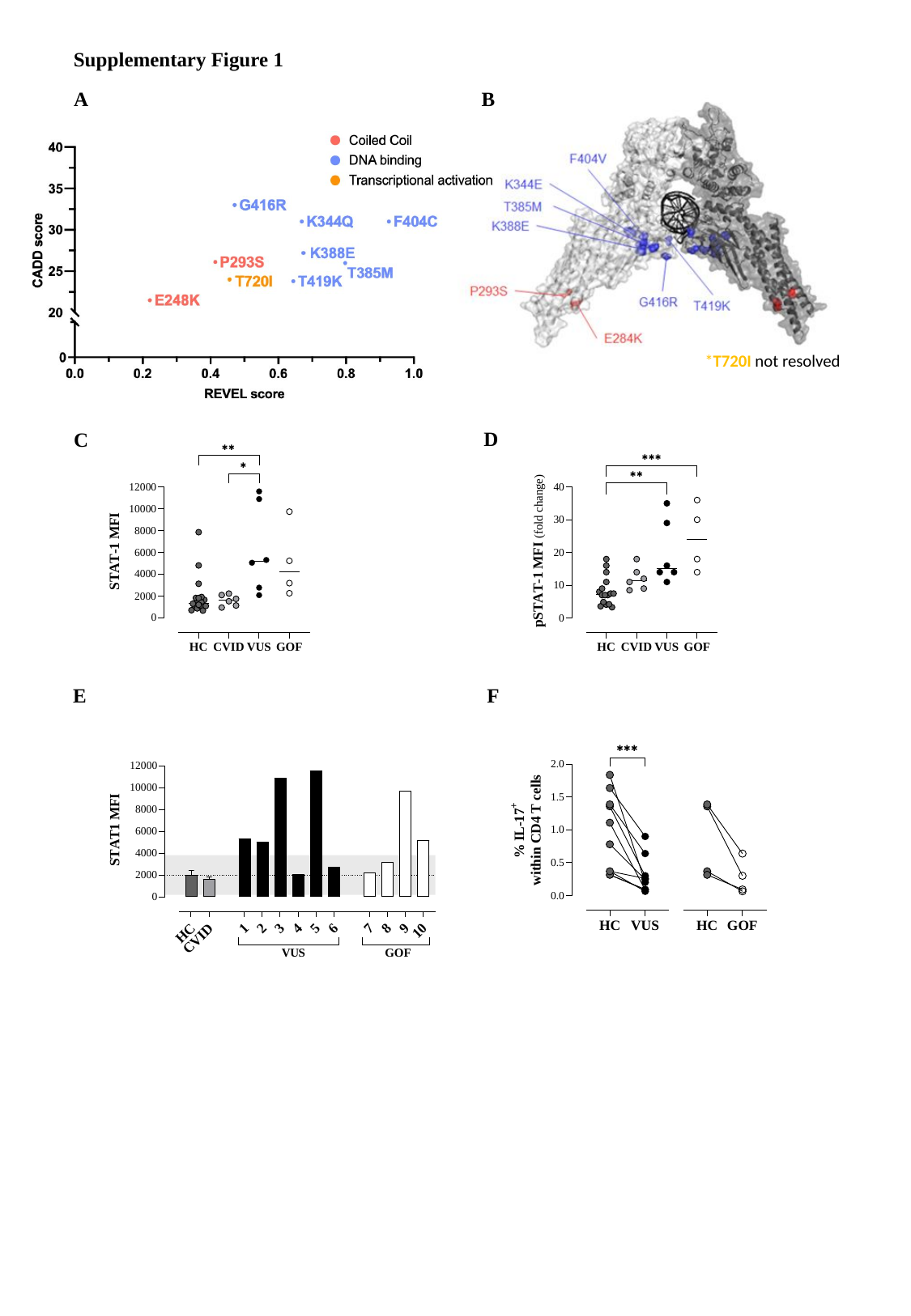

Supplementary Figure 1
A
B
*T720I not resolved
D
C
E
F

## Slide 2
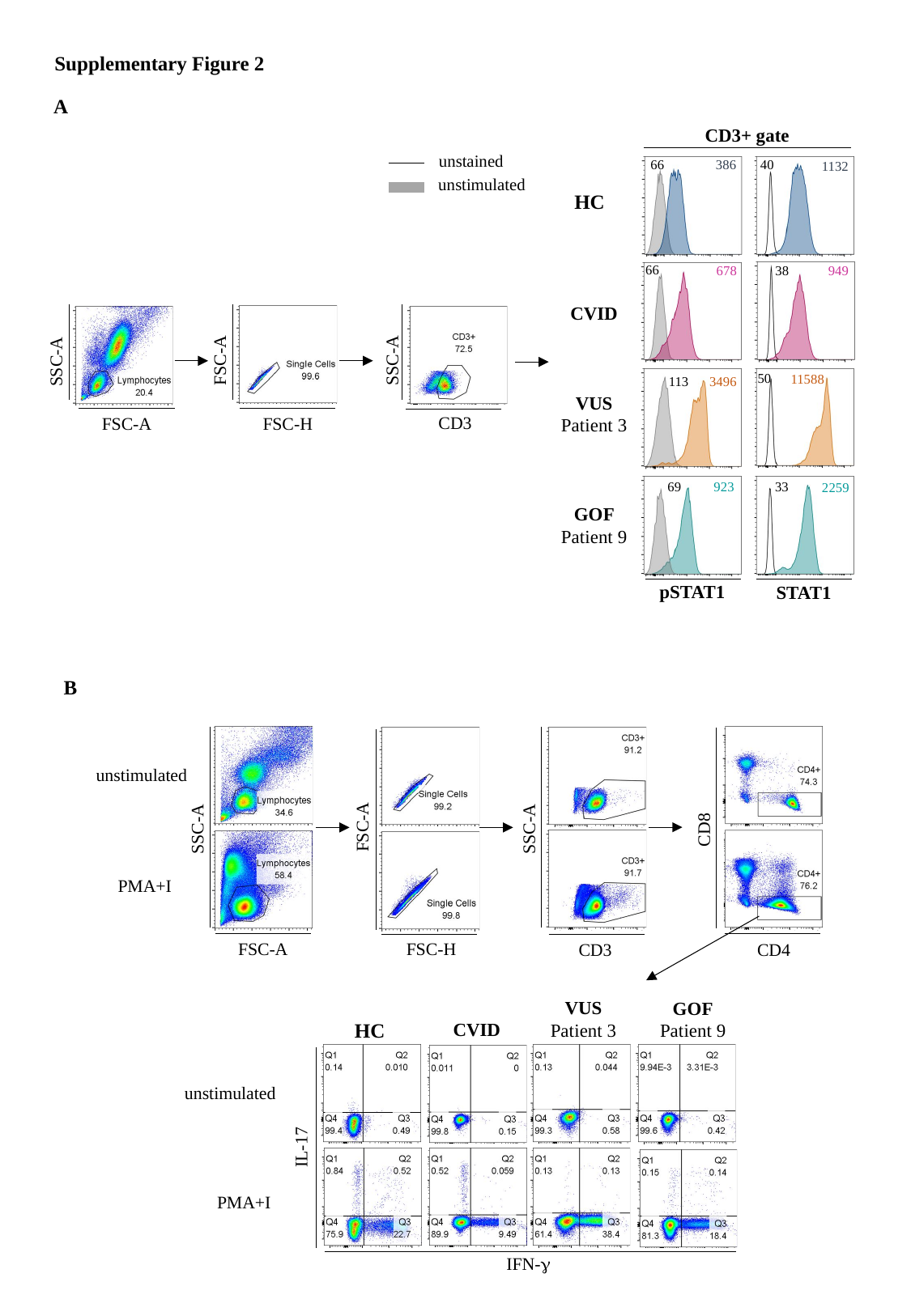

Supplementary Figure 2
A
CD3+ gate
unstained
unstimulated
66
40
386
1132
HC
66
38
949
678
CVID
FSC-A
SSC-A
SSC-A
50
11588
113
3496
VUS
Patient 3
CD3
FSC-A
FSC-H
69
923
33
2259
GOF
Patient 9
pSTAT1
STAT1
B
unstimulated
FSC-A
SSC-A
SSC-A
CD8
PMA+I
FSC-H
FSC-A
CD4
CD3
VUS
Patient 3
GOF
Patient 9
HC
CVID
unstimulated
IL-17
PMA+I
IFN-γ

## Slide 3
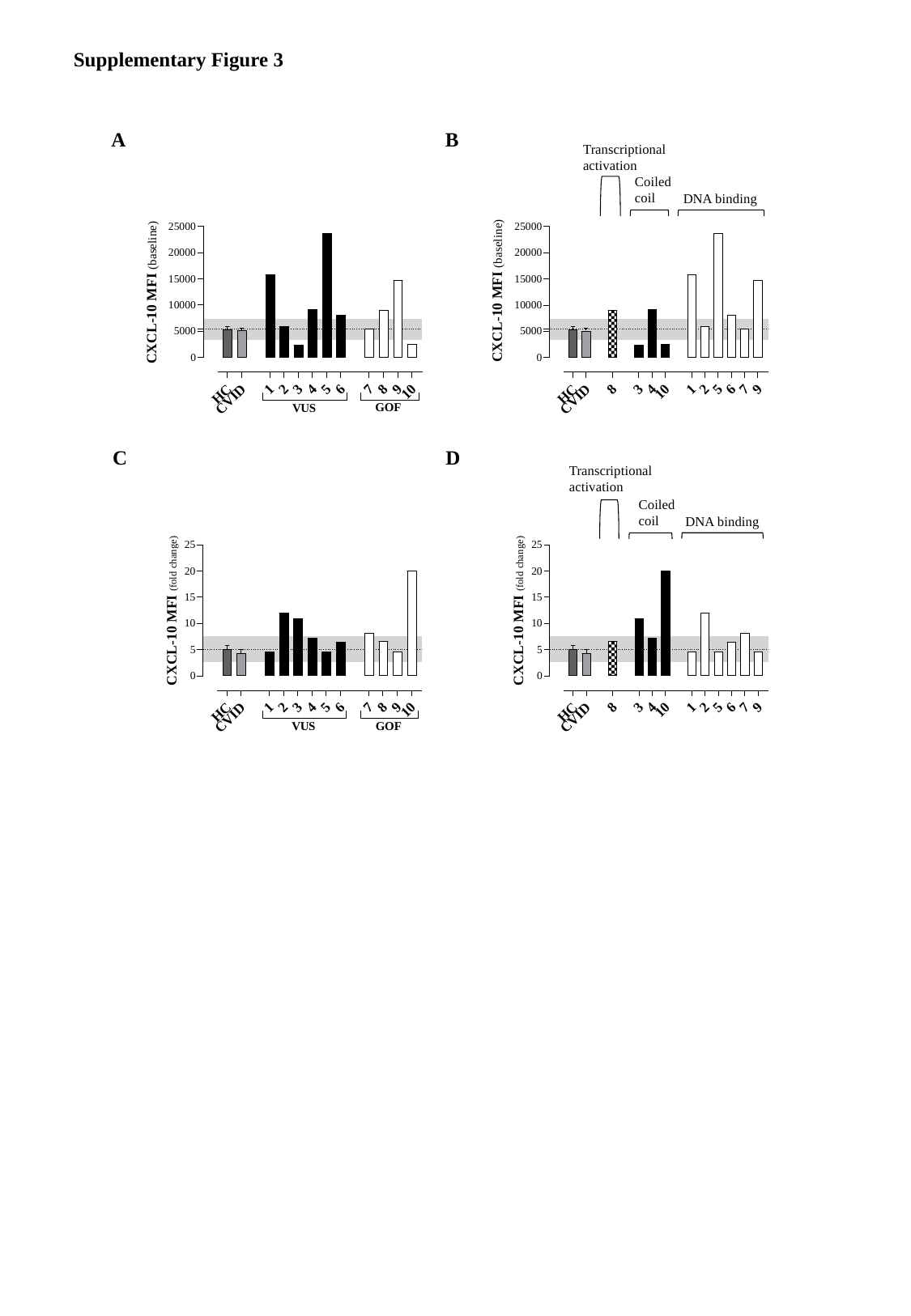

Supplementary Figure 3
A
B
Transcriptional
activation
Coiled
coil
DNA binding
C
D
Transcriptional
activation
Coiled
coil
DNA binding

## Slide 4
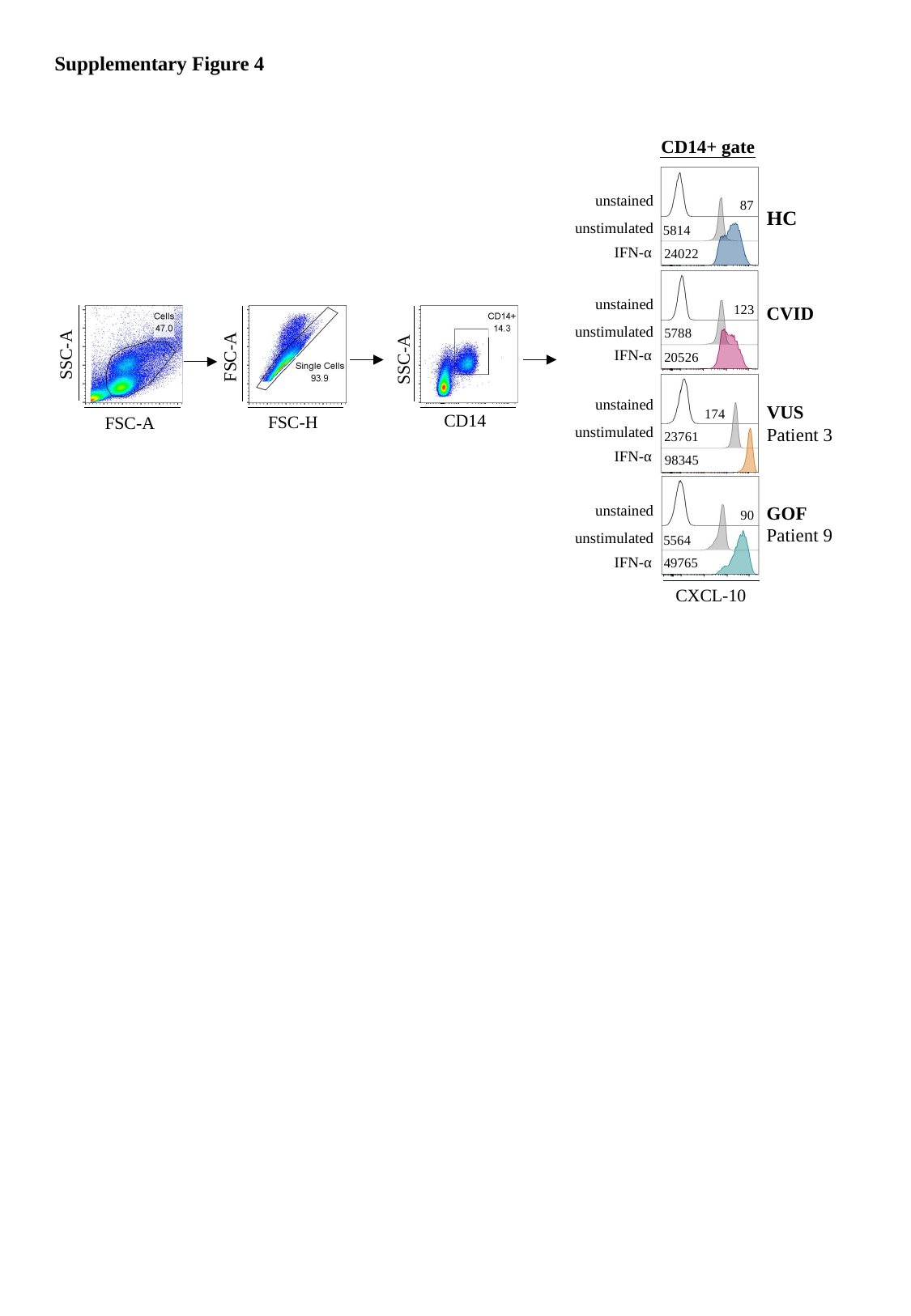

Supplementary Figure 4
CD14+ gate
unstained
87
HC
unstimulated
5814
IFN-α
24022
unstained
123
CVID
unstimulated
5788
IFN-α
20526
unstained
VUS
Patient 3
174
unstimulated
23761
IFN-α
98345
unstained
GOF
Patient 9
90
unstimulated
5564
IFN-α
49765
CXCL-10
SSC-A
FSC-A
SSC-A
CD14
FSC-H
FSC-A
