# Supplementary material for: Practical challenges for functional validation of STAT1 gain of function genetic variants
Source: Clin Exp Immunol. 2023 Feb 1;212(2):166–9. doi: 10.1093/cei/uxad008 (PMC10128160; doi:10.1093/cei/uxad008)
Supplement: uxad008_suppl_Supplementary_Table_S1 [file uxad008_suppl_supplementary_table_s1.docx]

**Supplementary Table 1: Clinical and immunological features of patients with *STAT1* VUS**

| Patient | 1 | 2 | 3 | 4 | 5 | 6 | 7 | 8 | 9 | 10 | |
| --- | --- | --- | --- | --- | --- | --- | --- | --- | --- | --- | --- |
| Sex | F | M | M | M | F | M | F | F | F | M | |
| Age (y) at onset | 3 | 16 | 3 | 1 | 3m | 23 | 15 | 2 | 6m | 15 | |
| Age (y) at diagnosis | 27 | 55 | 10 | 20 | 57 | 25 | 32 | 15 | 40 | 61 | |
| *STAT1* variant | E284K | E284K | K344Q | F404V | G416R | T419K | P293S | T385M | K388E | T720I | |
| Affected family | + | + | - | - | - | - | - | - | - | - | |
| Infections | | | | | | | | | | |  |
| CMC  Invasive | + | + | + | + | + | + | + | + | + | + | |
| fungal | - | - | - | - | - | - | - | - | - | - | |
| Chronic bacterial | - | - | Chest | Chest, folliculitis | Sinus | Chest | Chest | - | - | Folliculitis | |
| Mycobacterial | - | - | - | - | - | - | - | - | - | - | |
| Chronic viral | - | Shingles | - | - | - | Skin warts | Cervical warts HPV, HSV | - | - | - | |
| Chronic parasitic | - | Strongyloides | - | - | - | - | - | - | - | - | |
| Non-infectious complications | | | | | | | | | | |  |
| Auto-immunity | - | Scleroderma, Coeliac disease | - | - | Hypothyroidism, Sicca | - | - | - | - | - | |
| Auto-antibodies | - | GPC, TPO, ANA: speckled | - | - | ANA: centromere, TSH-R | - | ANA: cytoplasmic | - | - | ANA: nucleolar | |
| Bronchiectasis | - | - | + | + | + | + | + | + | - | - | |
| Malignancy | - | - | - | - | - | - | - | - | - | SCC tongue | |
| Allergy | - | Rhinitis | - | - | - | - | - | - | - | - | |
| Vasculopathy | - | - | - | - | - | - | - | - | - | - | |
| Other |  | Oral ulcers | GH insensitivity, splenomegaly, anaemia, thrombocytopaenia |  | Blepharitis |  |  |  |  |  | |
| Treatment | | | | | | | | | | |  |
| Fungal prophylaxis | + | + | + | + | + | + | + | + | + | + | |
| Antibiotic prophylaxis | - | - | Azithromycin | Azithromycin | - | - | Azithromycin | Azithromycin | - | - | |
| Ig therapy | - | - | + | - | - | - | - | + | - | - | |
| Immunosuppression | - | Azathioprine | Ruxolitinib | - | - | - | - | Tofacitinib | - | - | |
| HSCT | - | - | - | - | - | - | - | - | - | - | |
| Outcome | Alive | Alive | Alive | Alive | Alive | Alive | Alive | Alive | Alive | Alive | |
| Immunoglobulin level (g/L) | | | | | | | | | | |  |
| Age (y) at testing | 22 | 58 | 18 | 33 | 55 | 29 | 36 | 21 | 42 | 60 | |
| IgG | 18.9↑ | 19.1↑ | 10.9 | 9.3 | 12.6 | 7.1 | 10.7 | 8.3 | 16 | 13.6 | |
| IgA | 3.6 | 7.01↑ | <0.1↓ | 2.9 | 1.34 | 0.3↓ | 0.2↓ | 1.4 | 5.4↑ | 3.45 | |
| IgM | 1.24 | 0.15↓ | 1.8 | 1.2 | 2.69↑ | 1 | 2 | 1.2 | 2.26↑ | 0.55 | |
| Lymphocyte count x10^9/L | | | | | | | | | | |  |
| Total | 1.8 | 0.5↓ | 0.492↓ | 0.73↓ | 1.4 | 1.054 | 1.335 | 1.163 | U | 1.5 | |
| CD4+ | 0.948 | 0.375 | 0.210↓ | 0.26↓ | 0.648 | 0.307 | 0.615 | 0.729 | U | 0.796 | |
| CD8+ | 0.488 | 0.176↓ | 0.078↓ | 0.121↓ | 0.312 | 0.343 | 0.280 | 0.303 | U | 0.333 | |
| CD19+ | 0.208 | 0.027↓ | 0.141 | 0.343 | 0.363 | 0.211 | 0.139 | 0.033↓ | U | 0.158 | |
| CD16+ CD56+ | 0.101 | 0.054↓ | 0.015↓ | 0.093 | 0.198 | 0.103 | 0.15 | 0.033↓ | U | 0.235 | |
| y: year  m: months  CMC: Chronic mucocutaneous candidiasis  HPV: Human papillary virus  HSV: Herpes simplex virus  GPC: Gastric parietal cell antibodies  TPO: Thyroid peroxidase antibodies  ANA: Anti-nuclear antibodies  TSH-R: Thyroid stimulating hormone receptor antibodies | | | | | | | | | | | |
